# Evaluating the geographic distribution of cervical cancer patients presenting to a multidisciplinary gynecologic oncology clinic in Gaborone, Botswana

**DOI:** 10.1371/journal.pone.0271679

**Published:** 2022-08-04

**Authors:** Tara M. Friebel-Klingner, Hari S. Iyer, Doreen Ramogola-Masire, Lisa Bazzett-Matabele, Barati Monare, Alexander Seiphetlheng, Tlotlo B. Ralefala, Nandita Mitra, Douglas J. Wiebe, Timothy R. Rebbeck, Surbhi Grover, Anne Marie McCarthy

**Affiliations:** 1 Department of Biostatistics, Epidemiology and Informatics, Perelman School of Medicine, University of Pennsylvania, Philadelphia, Pennsylvania, United States of America; 2 Botswana-University of Pennsylvania Partnership, Gaborone, Botswana; 3 Dana-Farber Cancer Institute and Harvard TH Chan School of Public Health, Boston, Massachusetts, United States of America; 4 Department of Obstetrics and Gynecology, Faculty of Medicine, University of Botswana, Gaborone, Botswana; 5 Department of Obstetrics and Gynecology, Yale University, New Haven, Connecticut, United States of America; 6 Department of Oncology, Princess Marina Hospital, Gaborone, Botswana; 7 Department of Radiation Oncology, University of Pennsylvania, Philadelphia, Pennsylvania, United States of America; Wachemo University, ETHIOPIA

## Abstract

**Objective:**

In Botswana, cervical cancer is the leading cause of cancer death for females. With limited resources, Botswana is challenged to ensure equitable access to advanced cancer care. Botswana’s capital city, Gaborone, houses the only gynecologic oncology multi-disciplinary team (MDT) and the one chemoradiation facility in the country. We aimed to identify areas where fewer women were presenting to the MDT clinic for care.

**Methods:**

This cross-sectional study examined cervical cancer patients presenting to the MDT clinic between January 2015 and March 2020. Patients were geocoded to residential sub-districts to estimate age-standardized presentation rates. Global Moran’s I and Anselin Local Moran’s I tested the null hypothesis that presentation rates occurred randomly in Botswana. Community- and individual-level factors of patients living in sub-districts identified with higher (HH) and lower (LL) clusters of presentation rates were examined using ordinary least squares with a spatial weights matrix and multivariable logistic regression, respectively, with α level 0.05.

**Results:**

We studied 990 patients aged 22–95 (mean: 50.6). Presentation rates were found to be geographically clustered across the country (p = 0.01). Five sub-districts were identified as clusters, two high (HH) sub-district clusters and three low (LL) sub-district clusters (mean presentation rate: 35.5 and 11.3, respectively). Presentation rates decreased with increased travel distance (p = 0.033). Patients residing in LL sub-districts more often reported abnormal vaginal bleeding (aOR: 5.62, 95% CI: 1.31–24.15) compared to patients not residing in LL sub-districts. Patients in HH sub-districts were less likely to be living with HIV (aOR: 0.59; 95% CI: 0.38–0.90) and more likely to present with late-stage cancer (aOR: 1.78; 95%CI: 1.20–2.63) compared to patients not in HH sub-districts.

**Conclusions:**

This study identified geographic clustering of cervical cancer patients presenting for care in Botswana and highlighted sub-districts with disproportionately lower presentation rates. Identified community- and individual level-factors associated with low presentation rates can inform strategies aimed at improving equitable access to cervical cancer care.

## Introduction

Cervical cancer is a preventable malignancy affecting women worldwide. Mortality from cervical cancer is disproportionately higher in low-resource settings. In 2020, there were an estimated 604,000 new cervical cancer cases and 342,000 deaths, with the vast majority of deaths occurring in low resource settings, predominantly in sub-Saharan Africa (SSA) [1–3]. The majority of cervical cancer deaths in these settings can be attributed to poor access to prevention, screening, and the appropriate treatment [4–7].

In Botswana, an upper-middle-income country in SSA, cervical cancer is the most commonly diagnosed cancer and leading cause of cancer death for females [8]. The population of approximately 2.3 million people currently has over 700,000 women over the age of 20 at risk for developing cervical cancer [9]. The majority of cervical cancers are caused by Human Papilloma Viruses (HPV) [10], and co-infection of HPV and Human Immunodeficiency Virus (HIV) further increases cervical cancer risk [11, 12]. With the third highest HIV burden globally, cervical cancer prevention, screening, and treatment are a national priority in Botswana [13]. In recent decades the Botswana Ministry of Health and Wellness (MOHW) have adapted American Society of Clinical Oncology (ASCO) resource stratified screening strategies for its citizens, with the majority of cervical cancers being detected through loop electrosurgical excision procedure or visual inspection with acetic acid [13].

Botswana’s cancer control program is challenged with limited resources to ensure equitable access to advanced health care. A critical factor that affects cancer outcomes is a delay in a diagnosis resulting in the progression of a malignant tumor [14–16]. Thus, delayed access to care is essential when considering cancer morbidity and mortality. One aspect of access is geographic accessibility. Botswana is a sparsely populated country with only one clinic with a multi-disciplinary team (MDT) for gynecological oncology located in the capital city, Gaborone. The MDT clinic includes Botswana’s only gynecological oncologist and the one chemoradiation facility in the country capable of providing the standard of care, including staging and treatment, for cervical cancer patients diagnosed with locally advanced disease [17, 18].

Gaborone is located in the country’s southeast corner, over 1000 km away from the furthest residential areas. Geography, therefore, may be a significant barrier for accessing care [16]. Geographic information systems (GIS), which enables capturing and analyzing spatial and geographic data from a wide range of sources in conjunction with one another, is an efficient and robust methodical framework to investigate the role of geography and health in a population [19, 20]. The use of GIS technology and methods is rapidly evolving. GIS can help identify areas of limited access and help direct resources/planning efforts and illuminate factors that contribute to the bigger picture of public health. These methods can contribute to the limited cancer information available in low resource settings [21–23]. To our knowledge this is the first study to apply GIS methods to investigate the geographic distribution of cervical cancer patients presenting for care in Botswana.

The World Health Organization’s projected incidence of cervical cancer in Botswana for 2020 calculated using the Botswana National Cancer Registry [8] data from 2004–2008 is 374 cases [24]. Given one comprehensive MDT clinic where all women diagnosed with locally advanced cervical cancer should be referred to for treatment, we would anticipate over 300 cervical cancer cases to be referred to the MDT clinic per year. However, the MDT clinic has averaged less than 200 cases/year for the past five years (range: 96–256). We hypothesized that due to the lack of equitable access, not all women diagnosed with cervical cancer throughout Botswana are presenting to the MDT clinic for staging and treatment and that the rate of patients presenting for treatment is not random throughout Botswana. We applied GIS to identify areas with high or low presentation rates and highlight areas where suboptimal patterns of care exist. Highlighting areas with low presentation rates and understanding community- and individual-level characteristics associated with living in areas with low presentation rates can inform strategies for more equitable distribution of resources and highlight areas needing improved access to cancer treatment facilities.

## Materials and methods

### Study participants

This cross-sectional study included biopsy-proven cervical cancer patients referred to the MDT clinic between January 2015 and March 2020. The MDT clinic, located within Princess Marina Hospital (PMH), coordinates care for all gynecological oncology patients referred to the two tertiary hospitals in Gaborone: PMH and Gaborone Private Hospital (GPH) [18]. Patients who were over the age of 18, not pregnant, and were not diagnosed with cervical carcinoma in situ or recurrent disease, and had provided written, informed consent were included.

### Primary outcome measure

Our primary outcome was the rate of cervical cancer patients presenting to the MDT clinic for staging and/or treatment, henceforth referred to as ‘presentation rate’. To detect areas in Botswana with disproportionately high or low presentation rates, we defined our geographic unit of analysis as administrative sub-districts in Botswana. We used an available shapefile from the USCB [9] that defined the 2^nd^-order administrative boundaries for 28 sub-districts throughout the country. The USCB also defines 519 3^rd^ order administrative divisions described as “villages and associated localities” [9]. We abstracted each patient’s residential village from the questionnaire and geocoded each patient to one of USCB’s identified 519 villages [9].

To calculate the presentation rates, we aggregated cervical cancer patients per village and identified the appropriate administrative sub-district for each village. The denominator for each sub-district consisted of the total female population for patients 20 years and older from January 2015 to March 2020 [9]. We standardized the presentation rates using the overall Botswana female age distribution. Age-standardized presentation rates by sub-district were visually inspected using choropleth maps created in ArcGIS version 10.6.1 (Esri, Redlands, CA) for geographic variation.

### Covariates

Sub-district-level covariates included HIV prevalence, population density, and travel distance to the MDT clinic in Gaborone. The HIV prevalence for each sub-district was obtained from the Botswana AIDS Impact Survey IV [25]. The population density was calculated using the projected population relative to the polygon area per sub-district using projected estimates and the polygon area from the shapefile from the USCB [9]. Travel burden was determined using Google maps [26, 27] calculated as distance in kilometers traveled from the centroid of each sub-district to the MDT clinic in Gaborone.

A questionnaire was administered at the first visit to the MDT clinic. Data collected included patient sociodemographic and clinical factors. Sociodemographic factors included age, marital status, and village of residence; clinical factors included history of cervical cancer screening, ever/never visit with a traditional doctor and/or natural healer, presence of abnormal vaginal bleeding (including post-coital bleeding/bleeding after vaginal intercourse), and HIV status. Additional clinical data was abstracted from medical records for cervical cancer stage at presentation based on the International Federation of Gynecology and Obstetrics (FIGO) staging system [28, 29].

### Geographic analysis

To assess geographic patterns of presentation rates both nationally and locally, we employed Global Moran’s I and Anselin local index of spatial autocorrelation statistics, respectively [30, 31]. Global Moran’s I test was conducted to assess if presentation rates across the country were clustered, dispersed, or random (null hypothesis) and Anselin local index was used to investigate patterns of presentation rates for each sub-district [30, 31].

For Global Moran’s I, a positive spatial autocorrelation result indicates clustering, describing sub-districts with similar presentation rates within close geographic proximity to each other. Negative spatial autocorrelation reveals dispersion, noting sub-districts with dissimilar presentation rates were closer in geographical proximity in a manner that was not random. A null result indicates presentation rates across Botswana was random.

For the Global Moran’s I geographic analysis we defined neighbors using queen’s contiguity matrix [32] and conducted permutation tests with 999 simulations for significance. Using this weights matrix, the Global Moran’s I test enables presentation rates of each sub-district to be correlated with the mean presentation rates of neighboring sub-districts (spatial autocorrelation), thereby accounting for spatial dependence in presentation rates between sub-districts.

Next, using Anselin’s local index, we investigated patterns of presentation rates for each sub-district [30, 31]. This approach determines whether each individual (target) sub-district’s presentation rates are uniformly similar or disproportionately high or low relative to the mean presentation rates of that sub-district’s neighbors, once again defined by Queen’s contiguity matrix [32]. Permutation tests with 999 simulations were conducted and statistically significant clusters (similar presentation rates) or outliers (dissimilar presentation rates) were identified with 95% confidence intervals. A significant result revealed one of four possible categories for each sub-district, two types of clusters or two types of outliers. A high-high (HH) cluster indicated target sub-districts with high presentation rates were surrounded by neighboring sub-districts with high presentation rates. Conversely low-low (LL) clusters identified target sub-districts with low presentation rates surrounded by neighboring sub-districts with low presentation rates. High-low (HL) or low-high (LH) outliers indicated an inverse relationship between the target sub-district presentation rates and the mean of their neighbor’s presentation rates.

### Statistical analysis

Simple ordinary least squares (OLS) regression investigated the relationship of age-standardized presentation rates across the country with sub-district level community factors. If Moran’s I identified significant autocorrelation, we accounted for non-independence of presentation rates in our multivariable OLS regression model using a spatial weights matrix [33] to investigate associations with sub-district level variables (HIV prevalence, population density, and travel distance).

Lastly, we investigated individual-level clinical and demographic characteristics among cervical cancer cases living in a clustered (HH/LL) or outlier (HL/LH) identified sub-district compared to patients living in any other sub-district. Univariate individual level differences were assessed using student t-tests, chi-squared tests, and fishers exact tests as appropriate. Additionally, multivariable logistic regression models determined the magnitude of associations for patients living in identified LL or HH sub-districts versus patients not living in these sub-districts, while adjusting for demographic and clinical characteristics. Adjusted odds ratios and 95% confidence intervals with a cut-off α value of 0.05 determined significant associations. We performed geospatial analysis in ArcGIS version 10.6.1 (Esri, Redlands, CA) and open source GeoDa software [33]. We conducted all Statistical analysis in STATA 16.1 (College Station, TX).

### Ethics approval

The parent study, “Treatment and Outcomes of Patients Presenting with Cancer in Botswana,” was approved at the University of Pennsylvania as part of the Botswana-University of Pennsylvania Partnership (IRB: 820159 IRB#7 Penn) and by the Ministry of Health and Wellness of the Republic of Botswana (HPDME 13/18/1).

## Results

**Fig 1:**
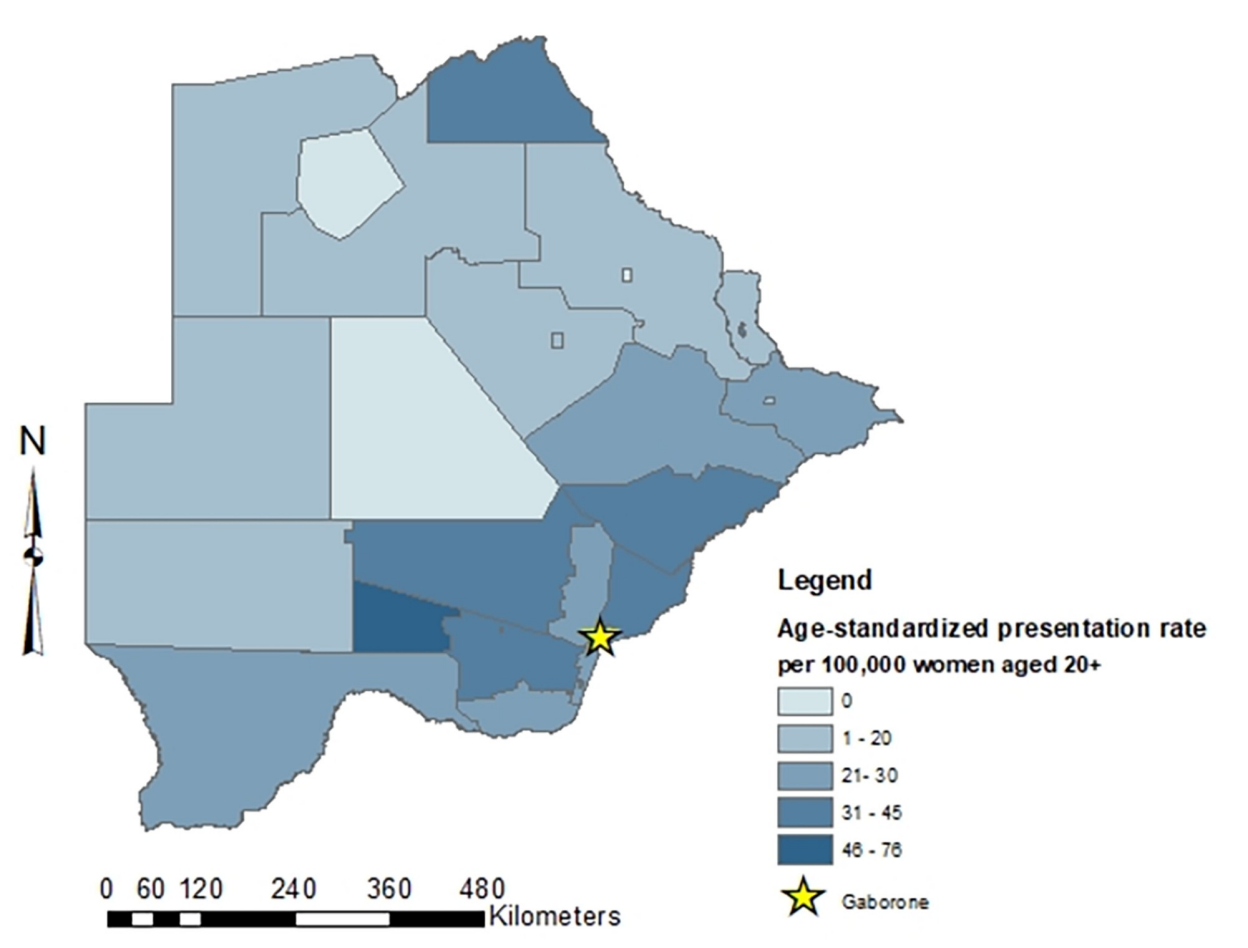
Age- standardized presentation rates to the MDT clinic in Gaborone, Botswana per sub-district*. *created in ArcGIS version 10.6.1 (Esri, Redlands, CA).

One thousand nineteen cervical cancer patients presented to the MDT clinic between January 2015 and March 2020. Nineteen patients (1.8%) diagnosed with cervical carcinoma in situ and 7 (0.7%) patients with recurrent disease were excluded. Thirty-four patients (3.3%) were missing village of residence, but 31 of these did have sub-district of residence and were geocoded to their respective sub-district. Thus, 990 cervical cancer patients were geocoded to one of the 28 sub-districts. Sociodemographic and clinical characteristics are shown in Table 1. Cervical cancer patients had a mean age of 50.6 years at time of presentation (range: 22.4–95.2). The overall presentation rate was 27.2 per 100,000 women (IQR: 12.5–35.0) for our study period. Age-standardized presentation rates by sub-districts are visually presented in a choropleth map (Fig 1). Crude and age-standardized presentation rates are presented in Table 2.

**Table 1:** Community and individual level characteristics of sub-districts and the study population.

***Community level factors***	N = 28	
	Mean	Standard Deviation
*Density per km* ^*2*^	290.74	644.09
*Travel distance (km)*	417.80	320.71
*HIV prevalence*	17.82	3.89
***Individual Level Factors***	N = 990	
	Mean	Range
*Age*	50.6	22.4–95.2
	Number	Percent
*HIV Positive*	680	69.9%
*Age of HIV positive (mean*, *range)*	*45*.*9*	*22*.*4–81*.*3*
*HIV Negative*	293	30.1%
*Age of HIV negative (mean*, *range)*	*60*.*5*	*27*.*4–95*.*2*
*Married/widowed/divorced*	339	34.2%
*Never married/single*	651	65.8%
*Early Stage (stage I/II)*	490	53.8%
*Late Stage (stage III/IV)*	420	46.2%
*Cervical cancer screening ever*	546	57.7%
*Cervical cancer screening never*	400	42.3%
*Visit with a traditional healer (Yes)*	98	10.2%
*Visit with a traditional healer (No)*	867	89.8%
*Abnormal vaginal bleeding (Reported)*	720	72.7%
*Abnormal vaginal bleeding (Not reported)*	270	27.3%

**Table 2:** Crude and age standardized presentation rates per 100,000 women.

Sub-district	Total Cases	Population (age 20+)	Crude rate per 100,000	Age-standardized rate per 100,000
Central Kgalagadi Game Reserve	0	138	0	0
Ngamiland Delta	0	5,399	0	0
Sowa Town	0	5,204	0	0
*Ngamiland West	10	96,743	9.30	9.49
*Ghanzi	7	70,513	9.93	10.62
Kgalagadi North	3	33,166	10.33	9.16
Selibe Phikwe	9	76,875	11.71	11.37
Tutume	39	248,989	15.66	13.51
*Ngamiland East	20	154,106	12.98	13.75
North East	19	107,059	17.75	16.07
Boteti	15	91,761	16.35	16.48
Orapa	2	15,783	12.67	19.56
South East	40	187,109	21.38	22.67
Gaborone	105	391,426	26.83	27.07
Serowe Palapye	95	205,953	31.05	28.07
Kweneng East	142	517,133	27.46	29.42
Bobonong	36	113,269	31.78	29.55
**Kgalagadi South	16	49,078	32.6	29.76
Barolong	31	88,936	34.86	30.91
Chobe	14	45,030	31.09	34.25
Jwaneng	6	30,305	19.8	34.5
Kweneng West	28	74,313	37.68	35.37
Lobaste	14	47,665	29.37	35.76
Mahalapye	75	187,537	39.99	35.94
Kgatleng	75	172,897	43.38	39.87
**Southern	102	217,937	46.8	41.16
Francistown	66	184,808	35.17	45.85
Ngwaketse West	21	23,062	91.06	75.98
**TOTAL**	990	3,635,573	27.23	27.23

*LL clustered sub-districts

**HH clustered sub-districts.

**Fig 2:**
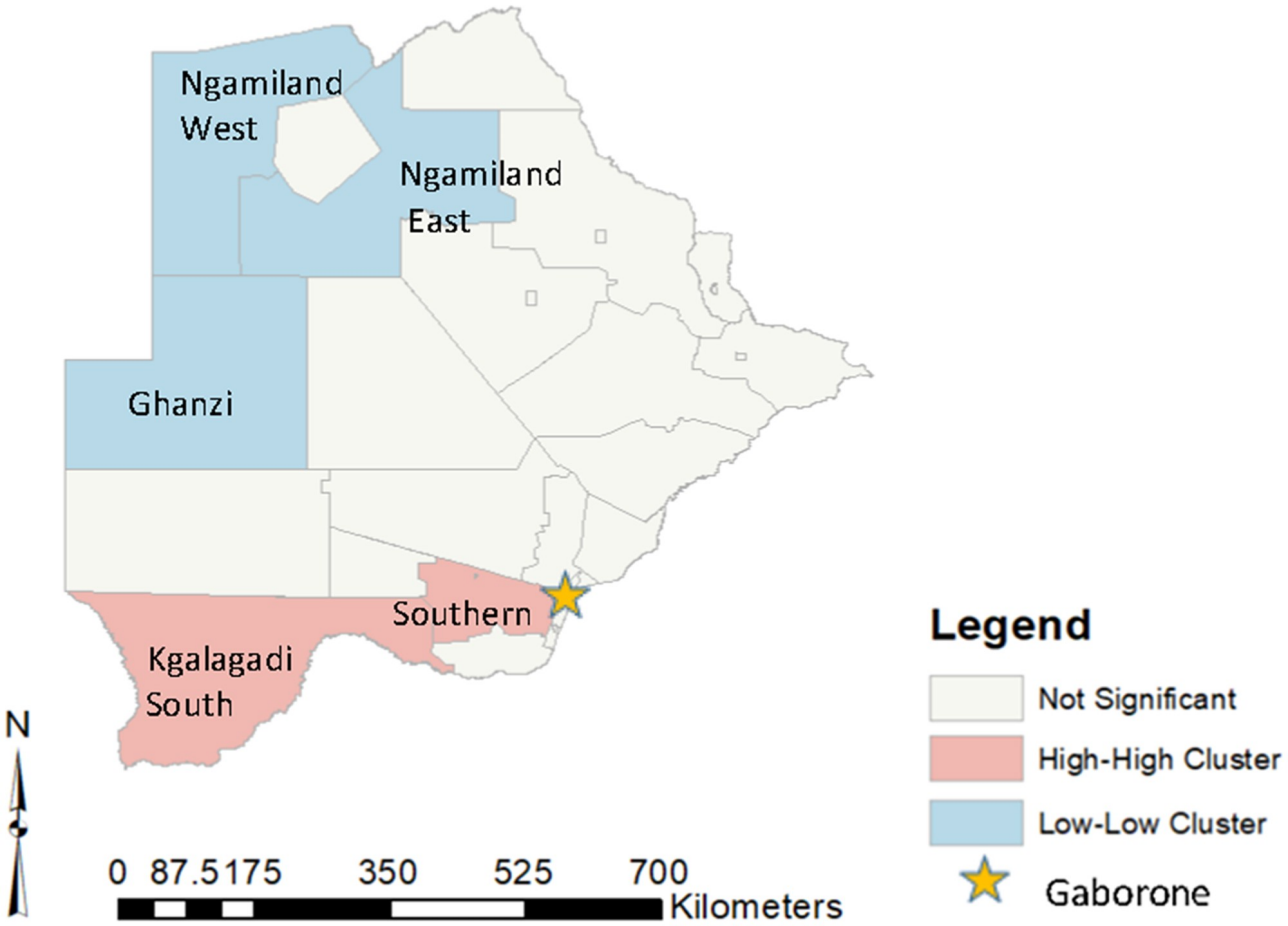
Identified High-High and Low-Low sub-district clusters*. *created in ArcGIS version 10.6.1 (Esri, Redlands, CA).

Positive spatial autocorrelation was observed (Global Moran’s I = 0.249; p = 0.014), identifying positive geographic clustering of cervical cancer rates across the country. Anselin local Moran’s I identified specific sub-districts where presentation rates were disproportionately high or low relative to their neighbors. Of the 28 sub-districts, five significant clustered (17.8%) sub-districts were identified, 2 HH and 3 LL sub-districts (Fig 2). No outliers (HL or LH sub-districts) were found. Two sub-districts were determined as HH clusters: Kgalagadi South and Southern (age-standardized rate per 100,000 women: 29.8 and 41.2, respectively). Three sub-districts were determined as LL clusters: Ngamiland West, Ngamiland East, and Ghanzi (age-standardized rate per 100,000 women: 9.5, 13.8, and 10.6 respectively).

Community-level factors for HH and LL sub-district clusters are shown in Table 3. HH sub-districts had a mean presentation rate of 35.5 per 100,000 women and LL sub-districts had a mean presentation rate of 11.33 per 100,000 women. Population density was 8.82 per km^2^ for HH sub-districts and 1.94 km^2^ for LL sub-districts. For HH sub-distritcs, the mean HIV prevalance for females was from 11.45 per 100,000 women and for LL sub-districts 15.27 per 100,000 women.

**Table 3:** Community level factors for identified High-High (HH) and Low-Low (LL) sub-districts.

Community level factors	HH sub-districts	Standard Deviation	LL Sub-districts	Standard Deviation
Number of sub-districts	2	--	3	--
Number of cases	118	--	37	--
presentation rates (mean)	35.5	7.78	11.33	2.52
HIV prevalence	11.45	0.49	15.27	1.8
Population density per km^2^	8.82	11.25	1.94	0.93
Travel distance (km)	341.5	255.27	912.0	274.97

Results from OLS regression showed a significant association between presentation rates across Botswana with travel distance (coefficient: -0.020, p = 0.034) to Gaborone, but no association with population density or HIV prevalence. Using the spatial weights matrix, multivariable OLS found that presentation rates across Botswana increased with decreased travel distance to Gaborone (adjusted coefficient: -0.026, p = 0.033). No significant associations were found for HIV prevalence or population density (Table 4).

**Table 4:** Results of OLS regression with spatial weights matrix for community level factors.

OLS regression	Coef	p-value	aCoef*	p-value
*HIV*	0.059	0.940	-0.084	0.912
*Population density*	1.75	0.560	-3.044	0.406
*Travel distance*	-0.020	0.034	-0.026	0.033

*adjusted coefficients.

Univariate individual-level differences for patients living in an LL sub-district were compared to not living in an LL sub-district and for patients living in a HH sub-district were compared to not living in a HH sub-district (Table 5). Women presenting from LL sub-districts were more commonly HIV positive (p = 0.025) and more often reported abnormal vaginal bleeding (p = 0.008) than women not presenting from LL sub-districts. Women presenting from HH sub-districts were older (p = 0.010), more commonly HIV negative (p<0.001), and more often presented with late-stage disease (p = 0.009) compared to women not presenting from HH sub-districts.

**Table 5:** Univariate analysis of Individual level factors for patients presenting from identified HH and LL sub-districts.

Individual Level Factors	Patients living in an LL Sub-district	Patients not living in an LL sub-district	p-value	Patients living in an HH sub-district	Patients not living in an HH sub-district	p-value
Sample Size(n)	37	953		118	872	
Age (mean (range))	47.4 (29.0–73.5)	50.7 (22.4–95.2)	0.12	53.4 (31.0–89.4)	50.2 (22.4–95.2)	0.010
	N (%)	N (%)		N (%)	N (%)	
HIV status						
Living with HIV	32 (86.5%)	648 (69.2%)	0.025	64 (55.7%)	616 (71.8%)	<0.001
Living without HIV	5 (13.5%)	288 (30.8%)		51 (44.3%)	242 (28.2%)	
Marital Status						
Married/widowed/divorced	14 (37.8%)	325 (34.1%)	0.640	43 (36.4%)	296 (33.9%)	0.590
Never married/single	23 (62.2%)	628 (65.9%)		75 (63.6%)	576 (66.1%)	
FIGO stage at diagnosis						
Early Stage (stage I/II)	18 (52.9%)	472 (53.9%)	0.910	47 (42.3%)	443 (55.4%)	0.009
Late Stage (stage III/IV)	16 (47.1%)	404 (46.1%)		64 (57.7%)	356 (44.6%)	
Cervical cancer screening						
Ever	21 (61.8%)	525 (57.6%)	0.630	66 (59.5%)	480 (57.5%)	0.690
Never	13 (38.2%)	387 (42.4%)		45 (40.5%)	355 (42.5%)	
Visit with a traditional healer						
Ever	3 (8.3%)	95 (10.2%)	0.710	12 (10.3%)	86 (10.1%)	0.940
Never	33 (91.7%)	834 (89.9%)		104 (89.7%)	760 (89.9%)	
Abnormal vaginal bleeding						
Reported	34 (91.9%)	686 (72.0%)	0.008	89 (75.4%)	631 (72.4%)	0.48
Not reported	3 (8.1%)	267 (28.0%)		29 (24.6%)	241 (27.6%)	

Additionally, multivariable logistic regression identified individual level factors associated with patients living in sub-districts with disproportionate presentation rates (Table 6). Patients living in LL sub-districts compared to patients not living in a LL sub-district more often reported experiencing abnormal vaginal bleeding (aOR: 5.88, 95% CI: 1.37–25.23) and were more likely to be HIV positive, though this did not reach statistical significance (aOR: 3.29; 95% CI: 0.97–11.17). These results should be interpreted with caution and considered exploratory due to the limited number of patients presenting from LL sub-districts. Patients living in HH sub-districts were less likely to be living with HIV relative to those not living in HH sub-districts (aOR: 0.46; 95% CI: 0.27–0.78) and more likely to be diagnosed with late-stage cervical cancer (aOR: 1.85; 95%CI: 1.19–2.87) compared to patients not living in a HH sub-district.

**Table 6:** Multivariable regression for individual level factors associated with patients presenting from identified HH and LL sub-districts.

	aOR	95% CI	p-value	aOR	95% CI	p-value
	LL sub-districts			HH sub-districts		
Age	1.00	0.96–1.03	0.798	1.00	0.98–1.02	0.956
HIV (Living with vs. without)	3.29	0.97–11.17	0.057	0.46	0.27–0.78	0.004*
Never married/single vs. married/widowed/divorced	0.50	0.23–1.08	0.077	0.98	0.61–1.56	0.918
FIGO stage						
(stage III/IV vs. I/II)	0.95	0.46–1.99	0.898	1.85	1.19–2.87	0.006*
Cervical cancer screening						
(Ever vs. never screened)	1.03	0.48–2.21	0.930	1.31	0.84–2.05	0.229
Visit with a traditional healer						
(Ever/never)	0.88	0.26–2.98	0.832	1.24	0.73–2.12	0.906
Abnormal vaginal bleeding						
Reported vs. Not reported	5.88	1.37–25.23	0.017*	1.24	0.73–2.12	0.422

aOR: adjusted Odds ratio

95% CI: 95% Confidence Interval

*p<0.05.

## Discussion

This study reveals non-random geographic patterns of patients diagnosed with cervical cancer throughout Botswana presenting to the MDT clinic in Gaborone. We identified specific areas of the country with disproportionately high and low presentation rates, indicating that areas with low presentation rates may have poor access to the one comprehensive care MDT clinic in Botswana. The areas with high presentation rates may have better access to the MDT clinic. Patients living in sub-districts with low presentation rates were more likely to present with abnormal vaginal bleeding, and patients living in sub-districts with high presentation rates were more likely to be HIV negative and more likely to present with late-stage disease. Identifying community and individual level factors associated with access to comprehensive cervical cancer care gives national health programs insight when developing strategies that target areas and populations being underserved by health care facilities. These strategies will be vital in preventing and controlling the emerging cervical cancer burden in Botswana.

In the literature, GIS methods have been used to examine local and regional variation of disease and to understand equity of access to care, as significant clustering of diseases may indicate potential inequity in access [34–36]. We carried out this study with the theory that geographic differences in presentation rates does not reflect the incidence or prevalence of cervical cancer but rather differences in patients accessing and presenting for treatment. Co-infection of HIV and HPV increases the risk of cervical cancer [11, 12], but our results identified no association between sub-district-level HIV prevalence and presentation rates across the country. This supports the hypothesis that the presentation rate is not likely a reflection of cervical cancer incidence or prevalence, but rather due inequity of access.

The literature has also reported that access to care is an important factor for cancer survival, particularly in low-resource settings [7, 37, 38]. Penchansky and Thomas [39] reported that access is multi-dimensional and consists of availability, accessibility, accommodation, affordability and acceptability. It could be inferred that sub-districts with high presentation rates have greater access than other sub-districts to care. One dimension of access is accessibility, or travel distance to the health center. Our results found that shorter/greater travel distance to the MDT clinic in Gaborone was associated with higher/lower presentation rates. Sub-districts in the Southern region of Botswana with closer proximity to Gaborone had increased presentation rates. In contrast, sub-districts with low presentation rates were identified in the northwest, furthest away and separated from Gaborone by the Central Kalahari Game Reserve, making travel to Gaborone much more challenging. Our study suggests that travel-distance accessibility to the MDT clinic in Gaborone presents a major barrier to care for cervical cancer patients in specific areas of the country.

Geographic accessibility is one factor impacting access to care. A more detailed understanding of this and other barriers of access in areas with low presentation rates could inform strategies to increase the number of cervical cancer cases presenting for care. Of note, sub-districts with higher presentation rates were not geographically the closest sub-districts to Gaborone, and Gaborone itself was not an area within a high-high cluster, further signifying that additional dimensions of access besides travel-distance accessibility are influencing access to care. For example, another barrier of access is lack of knowledge. Studies have shown that lack of knowledge regarding cervical cancer and cervical cancer screening as an identified barrier for cervical cancer care in Botswana; thus knowledge may differ according to geography [14, 40]. Tapela et. al. demonstrated a successful strategy implemented in Botswana at the healthcare level led to improved knowledge for primary care providers and reduced health system delays [41]. Understanding knowledge gaps in areas with low presentation rates could potentially increase cervical cancer patients presenting for care.

Previous studies in Botswana have shown that the lack of understanding of symptom severity is associated with a delay in seeking care [14, 40]. Patients in sub-districts with low rates often reported abnormal vaginal bleeding, indicating that they may not receive prompt medical attention and are not being referred until later stages when they are symptomatic. It may be beneficial to assess awareness of symptoms in areas with low presentation rates and areas with high presentation rates to understand if there is a difference at the provider or patient level in understanding abnormal vaginal bleeding as a symptom of cervical cancer. These findings also support previous reports to increase awareness, education, and screening for asymptomatic women in order to be able to detect cervical cancer at an earlier stage and improve outcomes [14, 40].

Our study also found that sub-districts with high presentation rates were associated with cervical cancer cases more likely to present at a late stage. While this may seem counterintuitive, due to the lack of comprehensive screening for asymptomatic women, cases throughout the country are more likely to be diagnosed at a late stage. Most studies have found that for SSA, including Botswana, more than half of cervical cancer cases are diagnosed at a late stage [5, 14, 42]. We could hypothesize that for sub-districts with higher access to Gaborone, patients diagnosed at a late stage when symptoms are present and morbidity is increased, may still be able to present to the MDT clinic for staging and treatment. However, with limited accessibility, patients diagnosed at a later stage further away from Gaborone may not be able to travel to the MDT clinic for treatment before succumbing to their illness. Thus, less late-stage cancers would be presenting from areas with low access. While our work does not directly address this inconsistency, future work could investigate the true prevalence of cervical cancer and the influence of morbidity in areas with low access or low rates.

In Botswana, cervical cancer screening and care have been implemented as part of the HIV care continuum [43]; thus, women living with HIV are likely to have increased health visits, more contact with health providers, and increased health literacy. Sub-districts with higher presentation rates had more HIV negative women present for treatment despite having no significant difference in community level HIV prevalence. A previous study also speculated that access to care may be differential according to HIV status in Botswana [44]. Thus, it is plausible that HIV negative women in sub-districts with lower presentation rates may be limited in cervical cancer awareness, screening, and contact with health care services. Increasing health literacy by developing and implementing educational programs, as well as increasing other health services, particularly in areas with low presentation rates and among women without HIV, could educate more women about the risks and symptoms of cervical cancer and the importance of screening and early detection to combat this disease. These efforts may increase the number of cervical cancers detected and increase the number of patients presenting for cervical cancer care.

Our study, the first to apply GIS to assess patterns of cervical cancer patients presenting for treatment in Gaborone, has limitations. It is important to note that due to the cross-sectional study design, no decisive conclusions can be made about the temporality or causality among the individual-level study variables. Also, those not seeking advanced medical care for symptoms of cervical cancer at the MDT clinic would not be captured in this cohort. The potential for selection bias would result in underrepresentation of cervical cancer in our dataset and the degree of selection bias could vary by geography, travel distance and other factors that could have impact on our results. For example, if patients with early-stage disease were being surgically treated for cervical cancer outside of the MDT clinic in areas with low presentation rates, this could bias our results towards the null and reject our hypothesis that less patients are presenting for care from these areas. In addition, community- and individual-level factors, such as poverty, socioeconomic status, education level and employment were not accounted for. These social determinants of health, likely to be geographically dependent, also influence various aspects of access. Future work to investigate these factors could contribute to a better understanding of the barriers of access in sub-districts with low presentation rates. Not accounting for these additional confounding or modifying factors could lead to a misinterpretation of our results. For example, if there is less awareness in areas further away from Gaborone, this lack of knowledge could explain the low presentation rates instead of travel distance. Future work accounting for additional dimensions of access is important to fully understand the barriers in areas with low presentation rates.

Our results reveal areas in Botswana with low presentation rates and where women living with cervical cancer may not be presenting for appropriate cervical cancer care. Our results also hypothesize about community- and individual-level factors that could be limiting access to health care for areas with low presentation rates. Our results indicated travel distance is a potential barrier to accessing cervical cancer care. We also identified that a potential area for intervention to mitigate the cervical cancer burden at the individual level could be to increase efforts for women without HIV, including assessing and improving knowledge in this population related to cervical cancer and cervical cancer screening. This finding is consistent with our previous studies [44, 45] indicating that access to care may be differential according to HIV status in Botswana. Our previous study [45] noted that WLWH were more likely to report having had cervical cancer screening (62% of WLWH reported cervical cancer screening versus 48% of women without HIV). Thus, future efforts to increase awareness campaigns and cervical cancer screening efforts to include women without HIV could be warranted. Additionally, early cervical cancer detection efforts should emphasize cancer symptom awareness, particularly abnormal vaginal bleeding, and educate women about the steps women should take if they experience symptoms.

## Conclusion

Botswana carries a heavy burden of cervical cancer. Access to adequate treatment is essential for early diagnosis and increased survival. Improving equitable access to cervical cancer treatment for women living in sub-districts with lower rates of cervical cancer patients presenting for care should be prioritized to help reduce the morbidity and mortality of cervical cancer.
